# Incision pressing, a simple and effective intervention to reduce colorectal surgical site infection: A propensity score-matched study

**DOI:** 10.3389/fsurg.2022.917559

**Published:** 2022-07-26

**Authors:** Yugang Jiang, Hongyuan Chen, Guotao Liu, Meifeng Liu, Meng Kong, Hongguang Sheng

**Affiliations:** ^1^ Department of Gastrointestinal Surgery, Shandong Provincial Hospital Affiliated to Shandong First Medical University, Jinan, China; ^2^ Department of Gastrointestinal Surgery, Shandong Provincial Hospital, Cheeloo College of Medicine, Shandong University, Jinan, China; ^3^ Departmet of General Surgery, Lanling People’s Hospital, Linyi, China

**Keywords:** surgical site infection (SSI), colorectal surgery, risk factor, prevention bundle, propensity score (PS) matching (PSM)

## Abstract

**Background:**

Colorectal surgery is associated with a high risk of surgical site infection (SSI). In March 2017, we developed an intervention, called “PRESS”, with the aim of reducing colorectal superficial SSI. This study assessed the effect of the new intervention in reducing the rates of superficial SSI in colorectal surgery.

**Methods:**

This study was a retrospective review of 312 PRESS+ patients compared to 171 historical control PRESS− patients who were 18 years of age or older and underwent elective colorectal surgery with clean-contaminated wounds from January 2015 to June 2020. In the PRESS+ groups, we pressed the incision downward hard with clean gauze after the interrupted suturing of the skin. Propensity score matching with 15 variables was performed in a 1:1 ratio to reduce selection bias. Univariate analysis and multivariate analysis were performed to identify risk factors associated with SSI.

**Results:**

The characteristics of the PRESS+ (*n* = 160) and PRESS− (*n* = 160) groups were well balanced after propensity score matching. The PRESS+ group had a lower superficial SSI rate (1.9% vs. 6.9%, *P* = 0.029) and a lower overall SSI rate (2.5% vs. 10.0%, *P* = 0.006) than the PRESS− group. Furthermore, multivariate analysis showed that the incisional press was an effective protective factor for superficial SSI (adjusted odds ratio = 0.215, 95% confidence interval = 0.057–0.818, *P* = 0.024). In addition, female sex (*P* = 0.048) and blood transfusion (*P* = 0.011) were demonstrated to be independent risk factors for superficial SSI.

**Conclusion:**

The incisional press after suturing is a simple, costless, and effective intervention in reducing superficial incisional SSI.

## Introduction

Surgical site infection (SSI) is a common postoperative complication after surgery (1). SSI leads to increased postoperative pain, longer hospital stays, increased healthcare costs and worse long-term survival outcomes (2, 3). Due to the high bacterial load in the colorectal lumen, colorectal surgery is associated with a high risk of SSI with incidence rates up to 34.7% (4–6).

To lower the incidence of SSI following colorectal surgery, many interventions have been studied as follows: mechanical bowel preparation; prophylactic oral and intravenous antibiotics; and appropriate skin preparation to reduce endogenous bacteria in the colorectal lumen and skin; wound protection; and subcutaneous wound irrigation to directly prevent wound contamination (5–10). Additionally, subcutaneous drainage is implemented to obliterate the dead space between the sutured skin and facia (11, 12). The presence of dead space has been believed to be a risk factor for superficial incisional SSI since the 1880s (13–15). However, the efficiency of subcutaneous drainage in reducing the rate of incisional SSI is controversial (12, 16–18).

In March 2017, we developed a simple and costless intervention, called “PRESS”, which could theoretically obliterate the incisional dead space. Here, we assessed the effect of this new intervention in reducing rates of superficial incisional infection in patients undergoing colorectal surgery. We also performed analyses to identify risk factors associated with SSI in our study population.

## Methods

### Description of intervention

**Figure 1:**
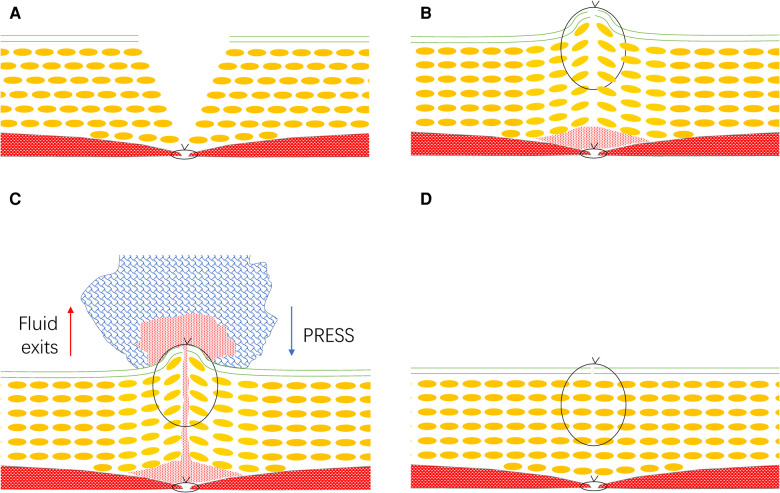
Illustration of suturing wound. Following closure of the linea alba fascia (**A**), the skin is closed with interrupted 2-0 nonabsorbable sutures without suturing subcutaneous tissue. As the stitches are tied, the skin and part of the subcutaneous tissue are usually gathered, creating a ridge in the middle of the incision, which causes the formation of dead space (**B**). The dead space accumulates tissue fluid and blood clots. To minimize dead space, we pressed the incision downward with clean gauze (**C**). The subcutaneous fat tissue was redistributed, and the dead space under the incision was obliterated. In general, tissue fluid seeped out from the dead space (**D**).

Suture often leads to the formation of dead space. Following continuous closure of the linea alba fascia with PDS Plus (Figure 1A), the skin is closed with interrupted 2-0 nonabsorbable sutures without suturing subcutaneous tissue. As the stitches are tied, the skin and part of the subcutaneous tissue are usually gathered, creating a ridge in the middle of the incision, which causes the formation of dead space between the subcutaneous tissue and sutured linea alba fascia (Figure 1B). The dead space accumulates tissue fluid and blood clots, facilitating the occurrence of SSI. To reduce the rate of superficial SSI, a unique intervention was developed in March 2017, which was performed on all the incisions in the subsequent colorectal surgeries performed at our institution. After completing the interrupted sutures of the skin, we pressed the incision downward hard with clean gauze using our hands (Figure 1C, and Supplemental Video), which resulted in a sensation of friction between the incisional tissues and stitches. The process of pressing took about half a minute to one minute, and was stopped until we could not feel any frictions. The subcutaneous fat tissue was redistributed, and the dead space under the incision was theoretically obliterated. In general, tissue fluid seeped out from the dead space (Figure 1D).

**Figure 2:**
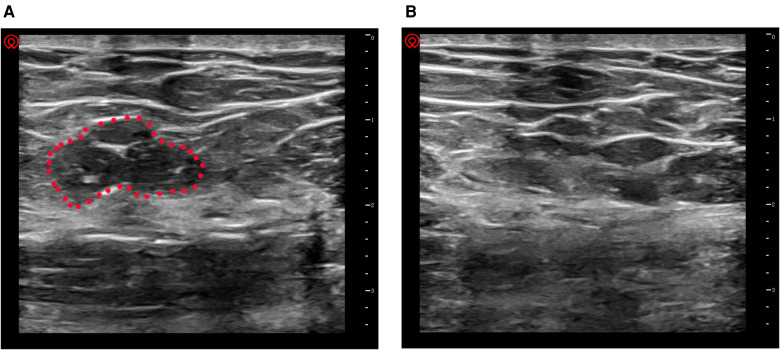
Verifying dead space by ultrasonography. (**A**) before incisional pressing. there was an obvious dead space under the incision (the area in the red dotted circle); (**B**) after incisional pressing, the dead space disappeared.

To verify the assumed effect of incisional pressing on obliterating the dead space, we examined the dead space under the incision before and after incisional pressing by ultrasonography. As shown in Figure 2, there was an obvious dead space (Figure 2A) before incisional pressing. Then the dead space was obliterated following incisional pressing in Figure 2B.

Before the introduction of the incisional press intervention, an SSI prevention bundle had been applied to patients who underwent colorectal surgery in our institution since January 2015, which included the following interventions: mechanical bowel preparation; prophylactic intravenous antibiotics; appropriate method of hair removal and skin preparation; application of wound edge protector; and wound irrigation.

In brief, oral polyethylene glycol electrolyte powder was administered on the day before surgery. Oral antibiotic bowel preparation was not performed. Second-generation cephalosporin and metronidazole were administered to all patients 30–60 min before surgery, repeated every 3 h during surgery or when 800 ml of estimated blood loss occurred and continued for 24 h after surgery. Hair removal was performed with clippers just before the surgery, and the skin was scrubbed with povidone–iodine three times and 75% alcohol one time. The midline surgical wound was protected by a plastic wound edge protector during laparotomy. After closure of the linea alba, the incision was routinely irrigated with 500 milliliter 0.9% saline. No subcutaneous suture was performed, and no subcutaneous drain was placed. Interrupted sutures with 2-0 Mersilk (Ethicon) were placed for skin closure. Finally, the incision was covered with sterile dressings in both groups. The incision was monitored every two days. A 30-day short-term follow-up was performed in the outpatient department by M.K. and Y.J.

### Study design and participants

The present study was a retrospective review of prospectively collected data from January 2015 to June 2020 in Shandong Provincial Hospital, China. Consecutive patients who were 18 years of age or older and underwent elective colorectal surgery with clean-contaminated wounds were included. Patients who underwent emergency laparotomy, abdominoperineal resection, Hartmann's procedure, colostomy and closure of stoma were excluded. We also excluded patients who were treated with steroids and who had bowel obstruction, perforation, any preoperative intraperitoneal infection and reoperation within 30 days due to nonwound complications. All the rectal cancer patients with neoadjuvant chemoradiotherapy were also excluded because of the existence of defunctioning stoma. This study was performed in line with the principles of the Declaration of Helsinki. The Ethical Committee of Shandong Provincial Hospital approved this study. Patient consent was waived because this was a retrospective review.

We divided the participants into the following two groups: (1) participants who received incisional press intervention from March 2017 to June 2020 (PRESS+ group); and (2) historical controls who did not receive incisional press intervention from January 2015 to February 2017 (PRESS− group).

### Variables and definitions of outcomes

Variables were collected directly from electronic patient records. Patient parameters, including sex, age, indication for surgery, body mass index (BMI), American Society of Anesthesiologists (ASA) score, smoking history, diabetes mellitus, cardiovascular diseases, hypertension, chronic obstructive pulmonary disease (COPD), preoperative hemoglobin (HGB), preoperative albumin (ALB), surgical approach (open or laparoscopic), surgical procedure (right hemicolectomy, left hemicolectomy, anterior resection or others), intraoperative estimated blood loss and perioperative blood transfusion, were analyzed. In the present study, the conversion from laparoscopic to open surgery was classified into open surgery, and sigmoid resection was classified into left hemicolectomy.

The primary outcome for our analysis was the incidence of superficial incisional SSI. SSIs were diagnosed by one of the experienced surgeons from our surgical team (M.K., C.H., Y.J. or H.S.) according to the Centers for Disease Control (CDC) guidelines (19). Superficial incisional SSI was considered as an infection that occurred within 30 days after the operation and involved only skin and subcutaneous tissue. The overall SSI, deep incisional SSI (involving only deep soft tissue) and organ/space SSI (involving only the intra-abdominal space) were analyzed separately. Anastomotic leakage (AL) was diagnosed according to the definition of the International Study Group of Rectal Cancer (20).

### Statistical analysis

Continuous variables are presented as the mean (standard deviation [SD]) or median (interquartile range [IQR]) depending on distribution type. To compare characteristics between groups, Student's t-test or Wilcoxon rank sum test was used for continuous variables, and Pearson's Chi-square test or Fisher’s exact test was used for categorical variables.

To estimate the impact of the incisional press on SSI with minimized selection bias between the PRESS+ group and the PRESS− group, propensity score matching was performed. Fifteen variables, including preoperative characteristics (age, sex, indication for surgery, BMI, smoking history, diabetes mellitus, cardiovascular diseases, hypertension, COPD, ASA score, preoperative CRT, preoperative ALB level and preoperative HGB level) and surgical characteristics (surgical approach and surgical procedure), were selected for matching. Optimal matching was performed in a 1:1 ratio without replacement and with a caliper distance of 0.03. A matched cohort was generated with well-balanced background characteristics.

Furthermore, in both the unmatched cohort and matched cohort, univariate analysis and multivariate analysis were performed sequentially to identify independent factors associated with superficial incisional SSI and overall SSI. Continuous variables were transformed into categorical variables for the logistic regression model. In particular, age greater than 65 years, BMI greater than or equal to 28, ASA score higher than or equal to 3, preoperative HGB level less than or equal to 110 and preoperative ALB level less than or equal to 35 were used as variables for the analysis. Variables with *P-*values <0.10 in the univariate analysis were then subjected to a multivariate stepwise backward logistic regression analysis. Values of the univariate and multivariate analyses were expressed as odds ratios (ORs) and 95% confidence intervals (CIs).

We used SPSS 24.0 (IBM Corp, Armonk, NY) for data analysis. R software 4.0.3 (R Project for Statistical Computing) was used to generate forest plots for the multivariate analysis results. All *P-*values were two-sided, and a value of *P* < 0.05 was considered statistically significant.

## Results

### Patient characteristics

**Figure 3:**
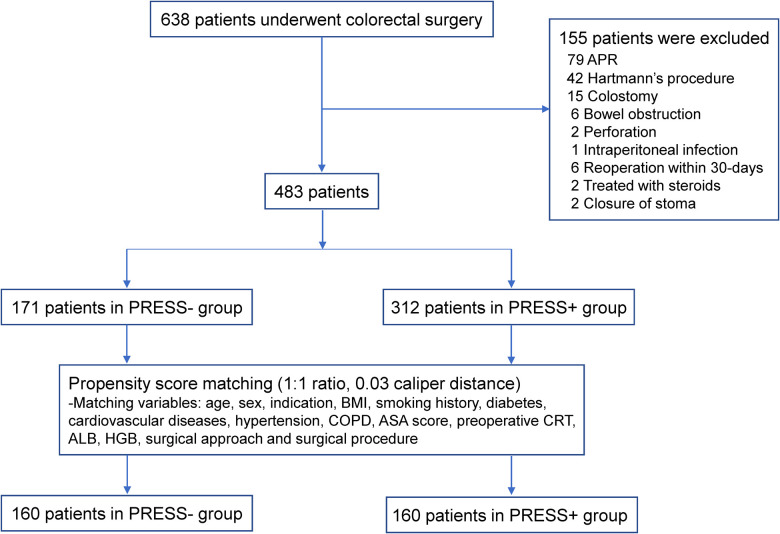
Flow diagram of the study. Abbreviations: APR, abdominoperineal resection; BMI, body mass index; COPD, chronic obstructive pulmonary disease; ASA, American Society of Anesthesiologists; CRT, chemoradiotherapy; ALB, albumin; HGB, hemoglobin.

We identified 638 patients who underwent elective colorectal surgery by our surgical team at the Shandong Provincial Hospital from January 2015 to June 2020. A total of 155 patients were excluded according to the exclusion criteria (Figure 3). A total of 483 patients were included in the analysis as follows: 312 patients received the incisional press procedure (PRESS+ group); and 171 patients who underwent surgery before March 2017 were assigned to the control group (PRESS− group). All the patients were available for a 30-day follow-up. Table 1 compares the preoperative and surgical characteristics between the PRESS+ and PRESS− groups. There were more patients with colorectal cancer (95.5% vs. 90.1%, *P* = 0.019) and laparoscopic surgery (67.0% vs. 36.8%, *P* < 0.001) in the PRESS+ group than in the PRESS− group. Other baseline characteristics were similar (*P* > 0.05) between groups before matching. After matching, 160 patients in each group remained for the final analysis. All preoperative and surgical characteristics, including indications for surgery (*P* = 0.671) and surgical approaches (*P* = 0.909), were well balanced between the groups.

**Table 1:** Comparison of baseline characteristics and surgical characteristics in the overall population, before and after propensity score matching.

Variables	All patients	Before matching			After matching		
		PRESS−group	PRESS + group	*P-*value	PRESS−group	PRESS+ group	*P-*value
	*n* = 483	*n* = 171	*n* = 312		*n* = 160	*n* = 160	
Patient characteristics							
Age, mean (SD), y	59.59 (12.14)	58.82 (11.98)	60.00 (12.22)	0.308	59.39 (11.57)	59.48 (12.68)	0.952
Sex							
Female	171 (43.3)	74 (43.3)	135 (43.3)	>0.99	69 (43.1)	70 (43.8)	0.910
Male	312 (56.7)	97 (56.7)	177 (56.7)		91 (56.9)	90 (56.3)	
Indication							
Colorectal cancer	452 (93.6)	154 (90.1)	298 (95.5)	**0**.**019**	147 (91.9)	149 (93.1)	0.671
Other	31 (6.4)	17 (9.9)	14 (4.5)		13 (8.1)	11 (6.9)	
BMI, mean (SD), kg/m^2^	24.37 (3.43)	24.65 (3.53)	24.23 (3.37)	0.225	24.62 (3.45)	24.44 (3.76)	0.679
Smoking history							
No	323 (66.9)	120 (70.2)	203 (65.1)	0.254	110 (68.8)	109 (68.1)	0.904
Yes	160 (33.1)	51 (29.8)	109(34.9)		50 (31.3)	51 (31.9)	
Diabetes mellitus							
No	412 (85.3)	147 (86.0)	265 (84.9)	0.760	138 (86.3)	138 (86.3)	>0.99
Yes	71 (14.7)	24 (14.0)	47 (15.1)		22 (13.8)	22 (13.8)	
Cardiovascular diseases							
No	445 (92.1)	157 (91.8)	288 (92.3)	0.847	148 (92.5)	148 (92.5)	>0.99
Yes	38 (7.9)	14 (8.2)	24 (7.7)		12 (7.5)	12 (7.5)	
Hypertension							
No	343 (71.0)	119 (69.6)	224 (71.8)	0.610	114 (71.3)	118 (73.8)	0.617
Yes	140 (29.0)	52 (30.4)	88 (28.2)		46 (28.8)	42 (26.3)	
COPD							
No	458 (94.8)	165 (96.5)	293 (93.9)	0.221	154 (96.3)	150 (93.8)	0.305
Yes	25 (5.2)	6 (3.5)	19 (6.1)		6 (3.8)	10 (6.3)	
ASA score							
I-II	348 (72.0)	123 (71.9)	225 (72.1)	0.965	115 (71.9)	110 (68.8)	0.541
III-IV	135 (28.0)	48 (28.1)	87 (27.9)		45 (28.1)	50 (31.3)	
Preoperative CRT							
No	418 (86.5)	147 (86.0)	271 (86.9)	0.783	137 (85.6)	142 (88.8)	0.403
Yes	65 (13.5)	24 (14.0)	41 (13.1)		23 (14.4)	18 (11.3)	
Preoperative HGB, mean (SD), g/L	125.49 (24.17)	125.89 (24.02)	125.27 (24.29)	0.787	125.50 (24.35)	123.76 (24.81)	0.526
Preoperative ALB, mean (SD), g/L	39.14 (4.16)	39.31 (4.04)	39.04 (4.24)	0.493	39.09 (3.95)	38.82 (4.46)	0.571
Surgical characteristics							
Surgical approach							
Open^a^	211 (43.7)	108 (63.2)	103 (33.0)	**<0**.**001**	97 (60.6)	98 (61.3)	0.909
Laparoscopic	272 (56.3)	63 (36.8)	209 (67.0)	** **	63 (39.4)	62 (38.8)	
Surgical Procedure							
Right hemicolectomy	134 (27.7)	47 (27.5)	87 (27.9)	0.500	45 (28.1)	48 (30.0)	0.956
Left hemicolectomy	88 (18.2)	33 (19.3)	55 (17.6)		30 (18.8)	32 (20.0)	
Anterior resection	248 (51.3)	84 (49.1)	164 (52.6)		80 (50.0)	75 (46.9)	
Others	13 (2.7)	7 (4.1)	6 (1.9)		5 (3.1)	5 (3.1)	
Blood Loss							
<200 ml	416 (86.1)	142 (83.0)	274 (87.8)	0.146	134 (83.8)	135 (84.4)	0.879
≥200 ml	67 (13.9)	29 (17.0)	38 (12.2)		26 (16.3)	25 (15.6)	
Blood transfusion							
No	413 (85.5)	146 (85.4)	267 (85.6)	0.953	136 (85.0)	131 (81.9)	0.452
Yes	70 (14.5)	25 (14.6)	45 (14.4)		24 (15.0)	29 (18.1)	

Abbreviations: SD, standard deviation; BMI, body mass index; COPD, chronic obstructive pulmonary disease; ASA, American Society of Anesthesiologists; CRT, chemoradiotherapy; HGB, hemoglobin; ALB, albumin.

Values in parentheses are percentages, unless identified otherwise.

Bold P-values indicate that differences between the groups were statistically significant.

^a^ Includes the procedures that are converted from laparoscopic to open surgery.

### Outcomes

The outcome parameters in the unmatched cohort and matched cohort are shown in Table 2. After matching, the PRESS+ group had a significantly lower overall SSI rate (2.5% vs. 10.0%, *P* = 0.006) and a significantly lower superficial incisional SSI rate (1.9% vs. 6.9%, *P* = 0.029) than the PRESS− group. However, the rates of other types of SSIs were not different between the two groups, including deep SSIs (0.0% vs. 0.6%, *P* = 1.000) and organ/space SSIs (0.6% vs. 2.5%, *P* = 0.371). Furthermore, wound disruption, anastomotic leakage and hospital stay did not significantly differ between the PRESS+ and PRESS− groups (*P* = 1.000, *P* = 0.556 and *P* = 0.136, respectively).

**Table 2:** Comparison of outcome parameters in the overall population, before and after propensity score matching.

Variables	All patients	Before matching			After matching		
		PRESS− group	PRESS+ group	*P-*value	PRESS− group	PRESS+ group	*P-*value
	*n* = 483	*n* = 171	*n* = 312		*n* = 160	*n* = 160	
Overall SSI							
No	458 (94.8)	154 (90.1)	304 (97.4)	**<0**.**001**	144 (90.0)	156 (97.5)	**0**.**006**
Yes	25 (5.2)	17 (9.9)	8 (2.6)		16 (10.0)	4 (2.5)	
Superficial SSI							
No	466 (96.5)	159 (93.0)	307 (98.4)	**0**.**002**	149 (93.1)	157 (98.1)	**0**.**029**
Yes	17 (3.5)	12 (7.0)	5 (1.6)		11 (6.9)	3 (1.9)	
Deep SSI							
No	482 (99.8)	170 (99.4)	312 (100.0)	0.354	159 (99.4)	160 (100.0)	>0.99
Yes	1 (0.2)	1 (0.6)	0 (0.0)		1 (0.6)	0 (0.0)	
Organ/space SSI							
No	476 (98.6)	167 (97.7)	309 (99.0)	0.251	156 (97.5)	159 (99.4)	0.371
Yes	7 (1.4)	4 (2.3)	3 (1.0)		4 (2.5)	1 (0.6)	
Wound disruption							
No	482 (99.8)	171 (100.0)	311 (99.7)	>0.99	160 (100.0)	159 (99.4)	>0.99
Yes	1 (0.2)	0 (0.0)	1 (0.3)		0 (0.0)	1 (0.6)	
AL							
No	466 (96.5)	164 (95.9)	302 (96.8)	0.612	153 (95.6)	155 (96.9)	0.556
Yes	17 (3.5)	7 (4.1)	10 (3.2)		7 (4.4)	5 (3.1)	
Hospital stay^a^, median (IQR), d	10 (8–11)	10 (8–13)	10 (8–11)	**0**.**019**	10 (8–13)	10 (9–11)	0.136

Abbreviations: SSI, surgical site infections; AL, anastomotic leakage; IQR, interquartile range.

Values in parentheses are percentages, unless identified otherwise.

Bold P-values indicate that differences between the groups were statistically significant.

### Factors associated with SSI

**Figure 4:**
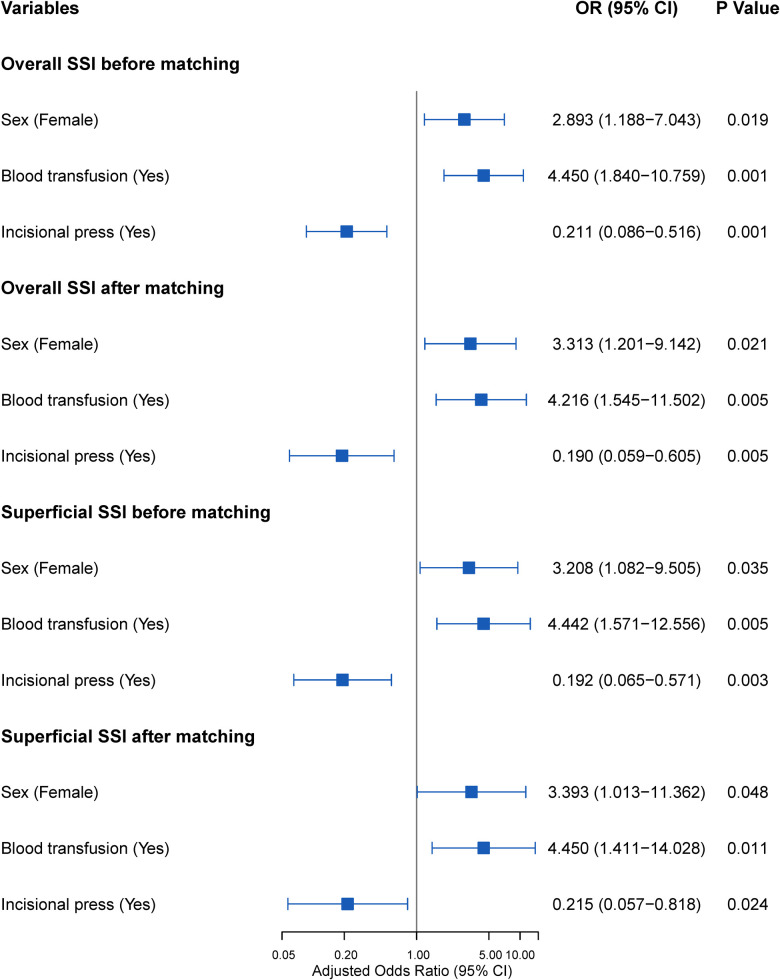
Adjusted odds ratios of overall SSI and superficial SSI before and after matching. Abbreviations: SSI, surgical site infection; OR, odds ratio.

Univariate analysis and multivariate analysis were performed successively to identify factors associated with overall SSI and superficial incisional SSI in our study. The results are presented in Figure 4, Supplementary Table S1, S2. Female sex and blood transfusion were significant independent risk factors for overall SSI and superficial SSI in both unmatched and matched cohorts. In contrast, incisional press was a significantly effective protective factor for overall and superficial SSI. In particular, the adjusted OR of superficial SSI was 3.393 for female sex (95% CI = 1.013–11.362, *P* = 0.048), 4.450 for blood transfusion (CI = 1.411–14.028, *P* = 0.011) and 0.215 for incisional press (95% CI = 0.057–0.818, *P* = 0.024) in the matched cohort.

## Discussion

SSI following colorectal surgery is a major cause of morbidity. To reduce the rate of SSI, we implemented a simple and costless intervention beyond the previously existing SSI prevention bundle in our institute. The propensity score-matched analysis indicated that implementation of an incisional press after suturing in elective colorectal surgery led to a significant reduction in superficial SSI and overall SSI. In addition, we demonstrated that incisional press intervention was a significantly effective protective factor, and female sex and blood transfusion were independent risk factors for superficial SSI and overall SSI.

Before the introduction of the incisional press procedure, an SSI prevention bundle was implemented in our institution, and the rate of superficial SSI was 6.9% in colorectal surgery, which was at a normal level compared with that in several previous studies (10, 21, 22). However, the SSI prevention bundle in our institution did not include interventions.

Dead space in the sutured wound may accumulate tissue fluid and blood clots, which are excellent culture media for possible bacteria from the colorectal lumen in the wound (14). Three strategies have been studied to obliterate the dead space as follows: suturing subcutaneous tissue and placing subcutaneous drains, and topical negative-pressure wound therapy. However, most of these studies did not find any benefits of suturing subcutaneous tissue on reducing SSI (23, 24). Holl et al. found that suture closure of the dead space increases the incidence of SSI (14), and they suggested that sutures may cause subcutaneous tissue necrosis, which may induce subcutaneous tissue loss and enlarge the dead space, eventually leading to wound infection. Moreover, the presence of stitches as foreign bodies may also increase the risk of bacterial infection.

Additionally, prophylactic subcutaneous drainage is used to decrease wound infection in many medical centers (11, 12). The placement of a subcutaneous drain could avoid wound fluid accumulation and eliminate the growth environment of bacteria in the dead space. However, the efficiency of subcutaneous drainage in reducing the rate of incisional SSI in clean-contaminated wounds is still controversial (16–18). Furthermore, placement of subcutaneous drains has several disadvantages in the enhanced recovery after surgery (ERAS) era as drains can cause pain and hinder early mobilization.

Moreover, as another method which could reduce fluid accumulation within the dead space (25, 26), topical negative-pressure wound therapy has been shown to be associated with reduced SSI rates of colorectal surgery in several studies (27, 28). However, the intervention is costly, and may cause skin-related complications, such as contact dermatitis (28).

In our present study, the incision pressing was first reported as an intervention with a theoretical effect on reducing the dead space. The incision pressing can not only force the tissue fluid out of the incision at the first time, but also avoid wound fluid accumulation in the incision in next few days. This intervention is easy to perform and requires one minute at most, and it does not cause postoperative pain or any inconvenience. A 5% reduction (6.9% to 1.9%) in the rate of superficial incisional SSI by this intervention was observed in our study. Furthermore, multivariate analysis also confirmed the protective role of the incisional press in superficial SSI with an odds ratio of 0.215. In summary, the incisional press after suturing is a simple, costless and effective intervention, suggesting that it should be used in colorectal surgery. However, given the study design, the effect of incisional press on obliterating dead space could not be precisely accessed and was more like a hypothetic mechanism. Further studies are needed to explore the specific mechanisms underlying the effect of incision pressing on reducing SSI.

Consistent with previous studies (29, 30), we identified female sex as an independent risk factor for superficial SSI. Due to estrogens, females have higher levels of subcutaneous adipose tissue than males (31). The thickness of subcutaneous fat tissue has been demonstrated to be positively associated with the incidence of SSI in colorectal surgery (32, 33), suggesting that females with thicker subcutaneous fat tissue may have higher risks of SSI. The multivariate analysis in the present study also demonstrated that perioperative blood transfusion increased the risks of superficial SSI and overall SSI, which agreed with previous findings on SSI in colorectal surgery (34–36). In the present study, patients with perioperative blood had a 4.216-fold higher risk of superficial incisional SSI than those without blood transfusion. Allogeneic blood transfusion may affect immunosuppression and increase the risk of infection following colorectal surgery (37, 38). Furthermore, the present findings that blood transfusion with no preoperative anemia was a risk factor highlighted the importance of minimizing blood loss in surgery.

There were several limitations to our study. First, this was a single-center retrospective study. Although 15 variables were included in the propensity score matching to reduce the effects of selective bias, other latent confounders that may have a role in the development of SSI may still exist. Therefore, further randomized trials are required to confirm the protective role of the incisional press in superficial SSI. Second, because more than 90% of patients in this study had colorectal cancer, this study did not represent patients with benign diseases, including inflammatory bowel disease and diverticular disease. Third, the skin was closed with interrupted sutures and without subcutaneous sutures in our study. Intervention with an incisional press may be only suitable for interrupted sutures rather than continuous subcuticular sutures or subcutaneous sutures. Fourth, midline incision was used for all the colorectal surgeries in this study. Therefore, whether incision pressing can be applied to other types of incisions needs further exploration. Finally, because the entire operative time did not reflect the time of incisional exposure in laparoscopic surgery and we lacked data about the time of surgical incision to skin closure, we did not include the operative time in the analysis.

## Conclusions

In conclusion, this study showed that incisional pressing after suturing is a simple, costless and effective intervention in reducing superficial incisional SSI. Thus, this intervention is suggested for colorectal surgery.

## Data Availability

The original contributions presented in the study are included in the article/**Suplementary Material**, further inquiries can be directed to the corresponding author/s.

## Supplementary material

Supplementary Video S1

How to press the incision.

## References

[1] Magill SS Edwards JR Bamberg W Beldavs ZG Dumyati G Kainer MA Multistate point-prevalence survey of health care-associated infections. N Engl J Med. (2014) 370:1198–208. 10.1056/NEJMoa1306801 24670166 PMC4648343

[2] Artinyan A Orcutt ST Anaya DA Richardson P Chen GJ Berger DH. Infectious postoperative complications decrease long-term survival in patients undergoing curative surgery for colorectal cancer: a study of 12,075 patients. Ann Surg. (2015) 261:497–505. 10.1097/SLA.0000000000000854 25185465

[3] Badia JM Casey AL Petrosillo N Hudson PM Mitchell SA Crosby C. Impact of surgical site infection on healthcare costs and patient outcomes: a systematic review in six European countries. J Hosp Infect. (2017) 96:1–15. 10.1016/j.jhin.2017.03.004 28410761

[4] Bennett-Guerrero E Pappas TN Koltun WA Fleshman JW Lin M Garg J Gentamicin-collagen sponge for infection prophylaxis in colorectal surgery. N Engl J Med. (2010) 363:1038–49. 10.1056/NEJMoa1000837 20825316

[5] Pinkney TD Calvert M Bartlett DC Gheorghe A Redman V Dowswell G Impact of wound edge protection devices on surgical site infection after laparotomy: multicentre randomised controlled trial (ROSSINI Trial). Br Med J. (2013) 347:f4305. 10.1136/bmj.f4305 23903454 PMC3805488

[6] Strobel RM Leonhardt M Krochmann A Neumann K Speichinger F Hartmann L Reduction of postoperative wound infections by antiseptica (RECIPE)?: a randomized controlled trial. Ann Surg. (2020) 272:55–64. 10.1097/SLA.0000000000003645 31599810

[7] Ikeda A Konishi T Ueno M Fukunaga Y Nagayama S Fujimoto Y Randomized clinical trial of oral and intravenous versus intravenous antibiotic prophylaxis for laparoscopic colorectal resection. Br J Surg. (2016) 103:1608–15. 10.1002/bjs.10281 27550722

[8] Espin Basany E Solís-Peña A Pellino G Kreisler E Fraccalvieri D Muinelo-Lorenzo M Preoperative oral antibiotics and surgical-site infections in colon surgery (ORALEV): a multicentre, single-blind, pragmatic, randomised controlled trial. Lancet. Gastroenterol Hepatol. (2020) 5:729–38. 10.1016/S2468-1253(20)30075-3 32325012

[9] Mihaljevic AL Schirren R Özer M Ottl S Grün S Michalski CW Multicenter double-blinded randomized controlled trial of standard abdominal wound edge protection with surgical dressings versus coverage with a sterile circular polyethylene drape for prevention of surgical site infections: a CHIR-Net trial (BaFO; NCT01181206). Ann Surg. (2014) 260:730–7; discussion 737–9. 10.1097/SLA.0000000000000954 25379844

[10] Koskenvuo L Lehtonen T Koskensalo S Rasilainen S Klintrup K Ehrlich A Mechanical and oral antibiotic bowel preparation versus no bowel preparation for elective colectomy (MOBILE): a multicentre, randomised, parallel, single-blinded trial. Lancet (London, England). (2019) 394:840–8. 10.1016/S0140-6736(19)31269-3 31402112

[11] Fujii T Tabe Y Yajima R Yamaguchi S Tsutsumi S Asao T Effects of subcutaneous drain for the prevention of incisional SSI in high-risk patients undergoing colorectal surgery. Int J Colorectal Dis. (2011) 26:1151–5. 10.1007/s00384-011-1228-2 21553008

[12] Watanabe J Ota M Kawamoto M Akikazu Y Suwa Y Suwa H A randomized controlled trial of subcutaneous closed-suction Blake drains for the prevention of incisional surgical site infection after colorectal surgery. Int J Colorectal Dis. (2017) 32:391–8. 10.1007/s00384-016-2687-2 27783162

[13] Mangram AJ Horan TC Pearson ML Silver LC Jarvis WR. Guideline for prevention of surgical site infection, 1999. Hospital Infection Control Practices Advisory Committee. Infect Control Hosp Epidemiol. (1999) 20:250–78; quiz 279–80. 10.1086/501620 10219875

[14] De Holl D Rodeheaver G Edgerton MT Edlich RF. Potentiation of infection by suture closure of dead space. Am J Surg. (1974) 127:716–20. 10.1016/0002-9610(74)90355-9 4598833

[15] Halsted WS. The treatment of wounds with special reference to the value of the blood clot in the management of dead space. IV. Operations for carcinoma of the breast by William S. Halsted, M.D., reprinted from Johns Hopkins Hospital Reports, Vol. II, No. 5, 277–280, 1891. CA Cancer J Clin. (1973) 23:96–8. 10.3322/canjclin.23.2.96 4196533

[16] Baier PK Glück NC Baumgartner U Adam U Fischer A Hopt UT. Subcutaneous Redon drains do not reduce the incidence of surgical site infections after laparotomy. A randomized controlled trial on 200 patients. Int J Colorectal Dis. (2010) 25:639–43. 10.1007/s00384-010-0884-y 20140620

[17] Pang K Sun P Li J Zeng N Yang X Jin L Prophylactic subcutaneous drainage reduces post-operative incisional infections in colorectal surgeries: a meta-analysis of randomized controlled trials. Int J Colorectal Dis. (2021) 36(8):1633–42. 10.1007/s00384-021-03908-8 33723634

[18] Coletta D Del Basso C Giuliani G Guerra F. Subcutaneous suction drains do not prevent surgical site infections in clean-contaminated abdominal surgery-results of a systematic review and meta-analysis. Langenbeck's Arch Surg. (2019) 404:663–8. 10.1007/s00423-019-01813-x 31468112

[19] Horan TC Andrus M Dudeck MA. CDC/NHSN surveillance definition of health care-associated infection and criteria for specific types of infections in the acute care setting. Am J Infect Control. (2008) 36:309–32. 10.1016/j.ajic.2008.03.002 18538699

[20] Rahbari NN Weitz J Hohenberger W Heald RJ Moran B Ulrich A Definition and grading of anastomotic leakage following anterior resection of the rectum: a proposal by the International Study Group of Rectal Cancer. Surgery. (2010) 147:339–51. 10.1016/j.surg.2009.10.012 20004450

[21] Mulder T Crolla R Kluytmans-van den Bergh MFQ van Mourik MSM Romme J van der Schelling GP Preoperative oral antibiotic prophylaxis reduces surgical site infections after elective colorectal surgery: results from a before-after study. Clin Infect Dis. (2019) 69:93–9. 10.1093/cid/ciy839 30281072

[22] Vo E Massarweh NN Chai CY Tran Cao HS Zamani N Abraham S Association of the addition of oral antibiotics to mechanical bowel preparation for left colon and rectal cancer resections with reduction of surgical site infections. JAMA Surg. (2018) 153:114–21. 10.1001/jamasurg.2017.3827 29049477 PMC5838711

[23] Paral J Ferko A Varga J Antos F Plodr M Lochman P Comparison of sutured versus non-sutured subcutaneous fat tissue in abdominal surgery. A prospective randomized study. Eur Surg Res. (2007) 39:350–8. 10.1159/000105263 17630491

[24] Gurusamy KS Toon CD Davidson BR. Subcutaneous closure versus no subcutaneous closure after non-caesarean surgical procedures. Cochrane Database Syst Rev. (2014) 24(1):Cd010425. 10.1002/14651858.CD010425.pub2 PMC11195627 24446384

[25] Kilpadi DV Cunningham MR. Evaluation of closed incision management with negative pressure wound therapy (CIM): hematoma/seroma and involvement of the lymphatic system. Wound Repair Regen. (2011) 19:588–96. 10.1111/j.1524-475X.2011.00714.x 22092797

[26] Mehdorn M Jansen-Winkeln B. Modified incisional negative pressure wound therapy increases seroma evacuation: an ex vivo model. Biomed Res Int. (2021) 2021:5846724. 10.1155/2021/5846724 34722767 PMC8553466

[27] Sahebally SM McKevitt K Stephens I Fitzpatrick F Deasy J Burke JP Negative pressure wound therapy for closed laparotomy incisions in general and colorectal surgery: a systematic review and meta-analysis. JAMA Surg. (2018) 153:e183467. 10.1001/jamasurg.2018.3467 30267040 PMC6583074

[28] Okuya K Takemasa I Tsuruma T Noda A Sasaki K Ueki T Evaluation of negative-pressure wound therapy for surgical site infections after ileostomy closure in colorectal cancer patients: a prospective multicenter study. Surg Today. (2020) 50:1687–93. 10.1007/s00595-020-02068-6 32638132

[29] Murray AC Pasam R Estrada D Kiran RP. Risk of surgical site infection varies based on location of disease and segment of colorectal resection for cancer. Dis Colon Rectum. (2016) 59:493–500. 10.1097/DCR.0000000000000577 27145305

[30] Pedroso-Fernandez Y Aguirre-Jaime A Ramos MJ Hernández M Cuervo M Bravo A Prediction of surgical site infection after colorectal surgery. Am J Infect Control. (2016) 44:450–4. 10.1016/j.ajic.2015.10.024 27038393

[31] Palmer BF Clegg DJ. The sexual dimorphism of obesity. Mol Cell Endocrinol. (2015) 402:113–9. 10.1016/j.mce.2014.11.029 25578600 PMC4326001

[32] Nakagawa H Ohno K Ikeda S Muto M. The effect of preoperative subcutaneous fat thickness on surgical site infection risk in patients undergoing colorectal surgery: results of a multisite, prospective cohort study. Ostomy Wound Manage. (2016) 62:14–20. 27564435

[33] Fujii T Tsutsumi S Matsumoto A Fukasawa T Tabe Y Yajima R Thickness of subcutaneous fat as a strong risk factor for wound infections in elective colorectal surgery: impact of prediction using preoperative CT. Dig Surg. (2010) 27:331–5. 10.1159/000297521 20689296

[34] Tang R Chen HH Wang YL Changchien CR Chen JS Hsu KC Risk factors for surgical site infection after elective resection of the colon and rectum: a single-center prospective study of 2,809 consecutive patients. Ann Surg. (2001) 234:181–9. 10.1097/00000658-200108000-00007 11505063 PMC1422004

[35] Xu Z Qu H Kanani G Guo Z Ren Y Chen X. Update on risk factors of surgical site infection in colorectal cancer: a systematic review and meta-analysis. Int J Colorectal Dis. (2020) 35:2147–56. 10.1007/s00384-020-03706-8 32748113

[36] Poon JT Law WL Wong IW Ching PT Wong LM Fan JK Impact of laparoscopic colorectal resection on surgical site infection. Ann Surg. (2009) 249:77–81. 10.1097/SLA.0b013e31819279e3 19106679

[37] Jensen LS Andersen AJ Christiansen PM Hokland P Juhl CO Madsen G Postoperative infection and natural killer cell function following blood transfusion in patients undergoing elective colorectal surgery. Br J Surg. (1992) 79:513–6. 10.1002/bjs.1800790613 1611441

[38] Heiss MM Mempel W Jauch KW Delanoff C Mayer G Mempel M Beneficial effect of autologous blood transfusion on infectious complications after colorectal cancer surgery. Lancet (London, England). (1993) 342:1328–33. 10.1016/0140-6736(93)92247-Q 7901637

